# NTRK fusion protein expression is absent in a large cohort of diffuse large B-cell lymphoma

**DOI:** 10.3389/fonc.2023.1146029

**Published:** 2023-03-14

**Authors:** Susanne Ghandili, Judith Dierlamm, Carsten Bokemeyer, Clara Marie von Bargen, Sören Alexander Weidemann

**Affiliations:** ^1^ Department of Oncology, Hematology and Bone Marrow Transplantation with Section Pneumology, University Cancer Center Hamburg, University Medical Center Hamburg-Eppendorf, Hamburg, Germany; ^2^ Institute of Pathology, University Medical Center Hamburg-Eppendorf, Hamburg, Germany

**Keywords:** diffuse large B-cell lymphoma, NTRK fusion protein, immunohistochemistry, tissue microarray, molecular landscape

## Abstract

**Background:**

Even though two NTRK-targeting drugs are available for the treatment of irresectable, metastatic, or progressive NTRK-positive solid tumors, less is known about the role of NTRK fusions in lymphoma. For this reason, we aimed to investigate if NTRK fusion proteins are expressed in diffuse large B-cell lymphoma (DLBCL) by systemic immunohistochemistry (IHC) screening and additional FISH analysis in a large cohort of DLBCL samples according to the ESMO Translational Research and Precision Medicine Working Group recommendations for the detection of NTRK fusions in daily practice and clinical research.

**Methods:**

A tissue microarray of 92 patients with the diagnosis of DLBCL at the University Hospital Hamburg between 2020 and 2022 was built. The clinical data were taken from patient records. Immunohistochemistry for Pan-NTRK fusion protein was performed and positive staining was defined as any viable staining. For FISH analysis only results with quality 2 and 3 were evaluated.

**Results:**

NTRK immunostaining was absent in all analyzable cases. No break apart was detectable by FISH.

**Conclusion:**

Our negative result is consistent with the very sparse data existing on NTRK gene fusions in hematologic neoplasms. To date, only a few cases of hematological malignancies have been described in which NTRK-targeting drugs may provide a potential therapeutic agent. Even though NTRK fusion protein expression was not detectable in our sample cohort, performing systemic screenings for NTRK fusions are necessary to define further the role of NTRK fusions not only in DLBCL but in a multitude of lymphoma entities as long as the lack of reliable data exists.

## Introduction

With an incidence of 7 cases per 100,000 persons per year, diffuse large B-cell lymphoma (DLBCL) is the most frequently observed histological type of non-Hodgkin lymphoma (NHL), accounting for approximately 25% of all NHL (1–3). DLBCL typically presents as rapidly enlarging lymphoma, commonly with extranodal involvement and/or constitutional symptoms (4). With current treatment approaches mainly consisting of combined therapy with CD20, CD79b-directed monoclonal antibodies, and conventional chemotherapy, DLBCL is curable in approximately 70% of patients (5). However, a lack of chemotherapy sensitivity must be assumed in patients with primary refractory or early relapsing DLBCL. To overcome the dismal prognosis of refractory or relapsed DLBCL, promising newly developed treatment approaches like CD19-directed CAR-T-cell therapies are already approved and available in selected countries or in the case of T-cell-engaging bi-specific antibodies currently investigated during multiple clinical trials (6–11). Nevertheless, despite the high overall response rates, the average time to CAR-T-cell reinfusion is approximately four weeks (12).

For this reason, the need for non-lymphodepleting chemotherapy-free bridging strategies is undisputed. In clinical routine, approved as well as off-label, individual bridging therapies with, for example, tafasitamab/lenalidomide, ibrutinib, or rituximab with polatuzumab vedotin are commonly used. In this setting, precision medicine based on the identification of driver mutations might provide additional treatment possibilities in a subset of DLBCL patients.

The neurotrophic tyrosine receptor kinase (NTRK) genes 1, 2, and 3 encode a tyrosine receptor kinase (TRK) receptor family (TRKA, TRKB, and TRKC), which plays an essential role during embryogenesis in the development of the nervous system and is moreover physiologically expressed in neuronal tissue (13–16). However, a gene rearrangement caused by a fusion of NTRK genes with different fusion partners can result in the development of TRK fusion oncoproteins which themselves lead to constitutive kinase activity or a simple overexpression of the kinase domain with subsequent activation of downstream cellular signaling pathways, which are involved in cell proliferation and survival (17, 18). NTRK fusions can occur with a high frequency (defined as > 25-35%) in a selected spectrum of rare pediatric cancers e.g. congenital infantile fibrosarcoma, congenital mesoblastic nephroma, secretory breast carcinoma or, mammary analog secretory carcinoma of the salivary gland or with an intermediate (>1% but < 25%) or low frequency (< 1%) in common adult solid cancers (19). The role of NTRK fusion genes has been extensively investigated and described in a broad spectrum of solid cancers leading to one of the first tissue-agnostic approvals of a highly effective targeted therapy by the Food and Drug Administration (FDA) when in 2018, larotrectinib was granted accelerated approval by the FDA as the first-in-class selective NTRK-inhibitor for the treatment of adult and pediatric patients with irresectable, metastatic, or progressive NTRK-positive solid tumors (20). However, the occurrence of NTRK-associated molecular findings in large cohorts of patients with hematological malignancies is rarely investigated. In 2020, Joshi et al. analyzed samples of 185 patients with acute myeloid leukemia, acute lymphoblastic leukemia, or myeloproliferative neoplasm. The authors identified a total of nine NTRK mutations, including four novel oncogenic NTRK point mutations potentially targetable by entrectinib (21). Recently, Witte et al. identified two cases of NTRK3 mutation in a cohort of 33 consecutive patients with plasmablastic lymphoma (22).

However, up to now, the occurrence of NTRK fusion protein expression or NTRK gene fusion in DLBCL has yet to be investigated. Since larotrectinib and entrectinib, two approved NTRK-targeted drugs are now available; we aimed to investigate the expression of NTRK fusion proteins in DLBCL by a systemic immunohistochemistry (IHC) screening and additional fluorescence *in situ* hybridization (FISH) analysis on a tissue microarray (TMA) containing samples from 92 DLBCLs from patients with newly diagnosed or refractory or relapsed (r/r) DLBCL according to the ESMO Translational Research and Precision Medicine Working Group recommendations for the detection of NTRK fusions in daily practice and clinical research (13).

## Materials and methods

### Patients

**Figure 1:**
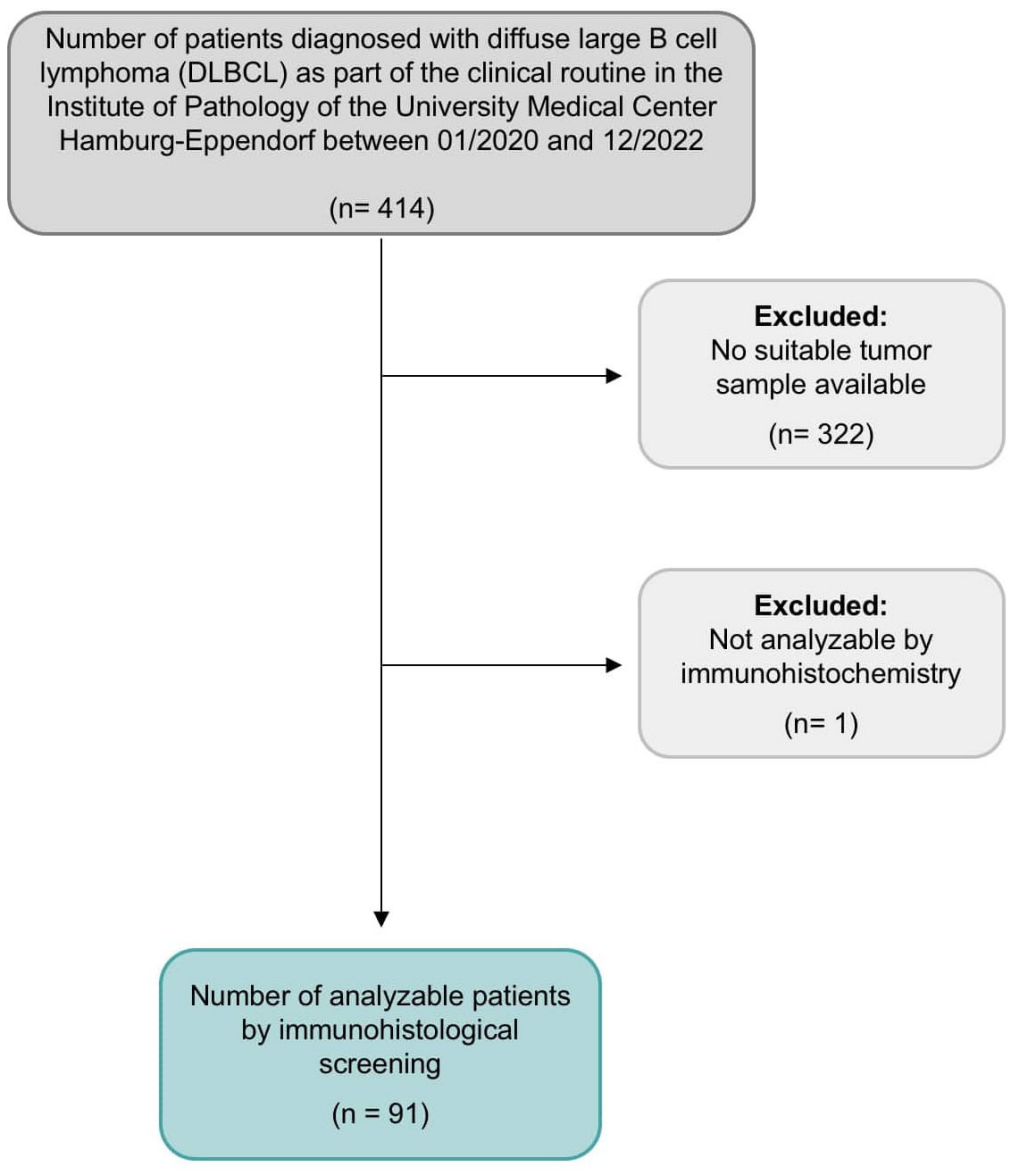
Flow chart of study design and population.

In this prospective observational, single-center analysis, we studied 92 consecutive patients with DLBCL, aged 18 years or older, who were immunohistochemically screened for an NTRK fusion gene protein overexpression. Samples of all patients included in this analysis were previously diagnosed with DLBCL as part of the clinical routine at the Institute of Pathology of the University Medical Center Hamburg-Eppendorf between 2020 and 2022. Patients

with high-grade B-cell lymphoma with MYC and BCL2 and/or BCL6 rearrangements were not excluded from this study. DLBCL was defined according to the 2016 revision of the World Health Organization classification of lymphoid neoplasms criteria (23). If available, clinical data regarding patients’ characteristics and DLBCL treatment were collected from the patient’s electronic medical records (**Figure 1**). The Ann Arbor classification was used for disease staging assessment (24). The International Prognostic Index (IPI) was used to calculate prognostic risk scores (25).

The data cut-off was on December 21, 2022. The use of archived remnants of diagnostic tissues for manufacturing of tissue microarrays and their analysis for research purposes as well as patient data analysis has been approved by local laws (HmbKHG, §12) and by the local ethics committee (Ethics Commission Hamburg, WF-049/09). All work has been carried out in compliance with the Helsinki Declaration.

### Tissue microarray (TMA)

The Institute of Pathology of the University Medical Center Hamburg-Eppendorf database includes 414 diagnoses of DLBCL for the years 2020 to 2022. Of these, 92 cases could be used to construct the TMA. The reasons for exclusion were mainly insufficient tissue quantities. Either because the tissue was already entirely or nearly completely utilized in the diagnostic workup or because it was a small needle biopsy containing too little material for tissue extraction for the TMA. Moreover, bone marrow biopsies are also generally unsuitable for TMAs. TMA construction was as previously described (26). In brief, tissue cylinders with a diameter of 0.6 mm each were taken from tumor-containing areas of selected “donor” tissue blocks and brought into empty recipient paraffin blocks. A large section of cerebral tissue was used as an on-slide positive control (27).

### Immunohistochemistry (IHC)

Freshly prepared TMA sections were immunostained on one day in one experiment. Slides were deparaffinized and exposed to heat-induced antigen retrieval for 5 minutes in an autoclave at 121°C in pH 9 Dako Target Retrieval Solution buffer (Dako, Glostrup, Denmark). Primary Anti-Pan Trk antibody (rabbit monoclonal, EPR17341, Abcam, Cambridge, MA) was applied according to the manufacturer’s instructions. The antibody reacts with a conserved proprietary peptide in the C-terminal part of TRK A, B and C. Bound antibody was then visualized using the EnVision Kit (Dako, Glostrup, Denmark) according to the manufacturer’s instructions. The percentage of NTRK-positive tumor cells was estimated, and the staining intensity was semi-quantitatively recorded (0, 1+, 2+, 3+). For statistical analyses, the staining results were categorized into four groups: Negative: no staining at all, weak staining: staining intensity of 1+ in ≤ 70% or staining intensity of 2+ in ≤ 30% of tumor cells, moderate staining: staining intensity of 1+ in > 70%, staining intensity of 2+ in > 30% but in ≤ 70% or staining intensity of 3+ in ≤ 30% of tumor cells, strong staining: staining intensity of 2+ in > 70% or staining intensity of 3+ in > 30% of tumor cells.

### Fluorescence *in situ* hybridization (FISH)

Freshly prepared TMA sections were pretreated (dewaxing, proteolysis) according to the instructions for use of the ZytoLight FISH-tissue Implementation Kit (Zyto Vision, Bremerhaven, Germany). 10 µl of SPEC NTRK1/NTRK2/NTRK3 Dual Color Break Apart Probe (Zyto Vision, Bremerhaven, Germany) was applied, posttreated and interpreted according to the manufactures manual. Hybridization quality was assessed semiquantitatively on a 1 - 3 scale (1=poor, 2=moderate, 3=good). Only results with quality 2 and 3 were evaluated.

### Endpoints

The primary aim of this study was to evaluate the rate of immunohistochemical expression of NTRK fusion protein in tumor samples of patients with newly diagnosed or r/r DLBCL.

### Statistical analysis

All statistical analyses were performed using Microsoft Excel for Mac, version 16.38 (Microsoft Cooperation, Redmon, Washington USA). Continuous values are presented as median. Nominal variables are expressed as numbers (%).

## Results

### Patients’ characteristics

A total of 92 samples of 92 consecutive patients diagnosed with DLBCL were included in the analysis. The most frequent tissue samples originated from lymph nodes (38%), followed by the testis (9%). The remaining samples originated from other extranodal tissues, including the small intestine, colon, skin, spleen, liver, buccal mucosa, and nasal mucosa. Patients’ demographics and baseline characteristics are presented in **Table 1**. The median age was 74 years (range 32-90), and the majority of patients were male (60%). In 41 (45%) patients, a germinal center B-cell-like cell of origin type was observed. Epstein-Barr-virus positivity was detectable in four patients (4%). Double hit expression was detectable in two patients (2%), whereas MYC-translocation was not assessed in 14 patients. IPI risk stratification showed high-risk DLBCL with IPI 4 or 5 in of 30 evaluable patients (17%, IPI was not assessable in 62 patients). In 13 patients, DLBCL transformed out of indolent lymphoma, including follicular lymphoma, lymphoplasmacytic lymphoma, chronic lymphatic lymphoma, and one case of angioimmunoblastic T-cell lymphoma.

**Table 1:** Patients’ characteristics.

Total number of analyzable patients, n	91
Age at DLBCL diagnosis, median (range)	74 (32-90)
Female sex, n (%)	37 (40)
Subtypes, n (%)	
Cell of origin type: Germinal enter B-cell-like	41 (45)
Epstein-Barr-virus positivity	4 (4)
Double hit	2 (2)
Triple hit	0
Ann Arbor stage, n (%)	
1A or 1B and 2A or 2B	18 (20)
3A or 3B	5 (5)
4A or 4B	14 (15)
Not evaluable	59 (65)
IPI, n (%)	
0-1	12 (13)
2-3	13 (14)
4-5	4 (4)
Not evaluable	62 (68)

### Immunohistochemical findings

**Figure 2:**
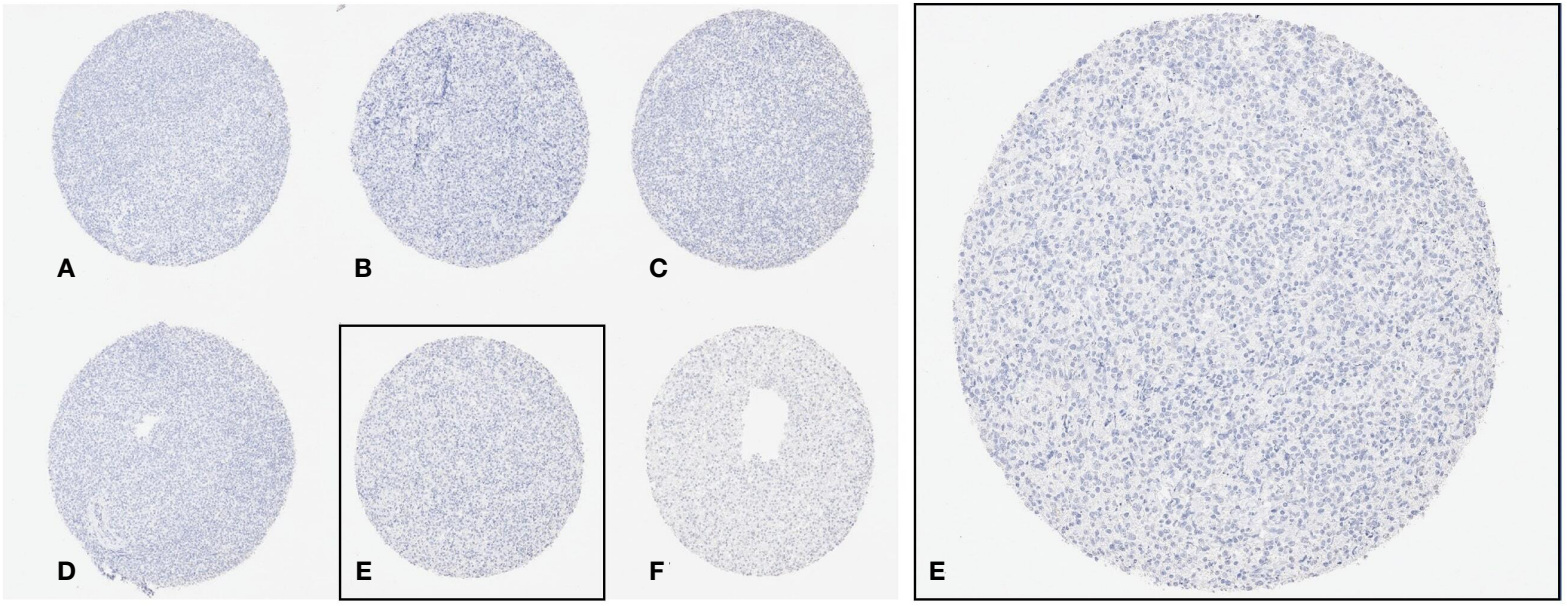
Left (40x): Overview of six spots from the TMA. Right (100x): Magnification of the spot e. **(A–F)** shows six exemplary TMA spots.

**Figure 3:**
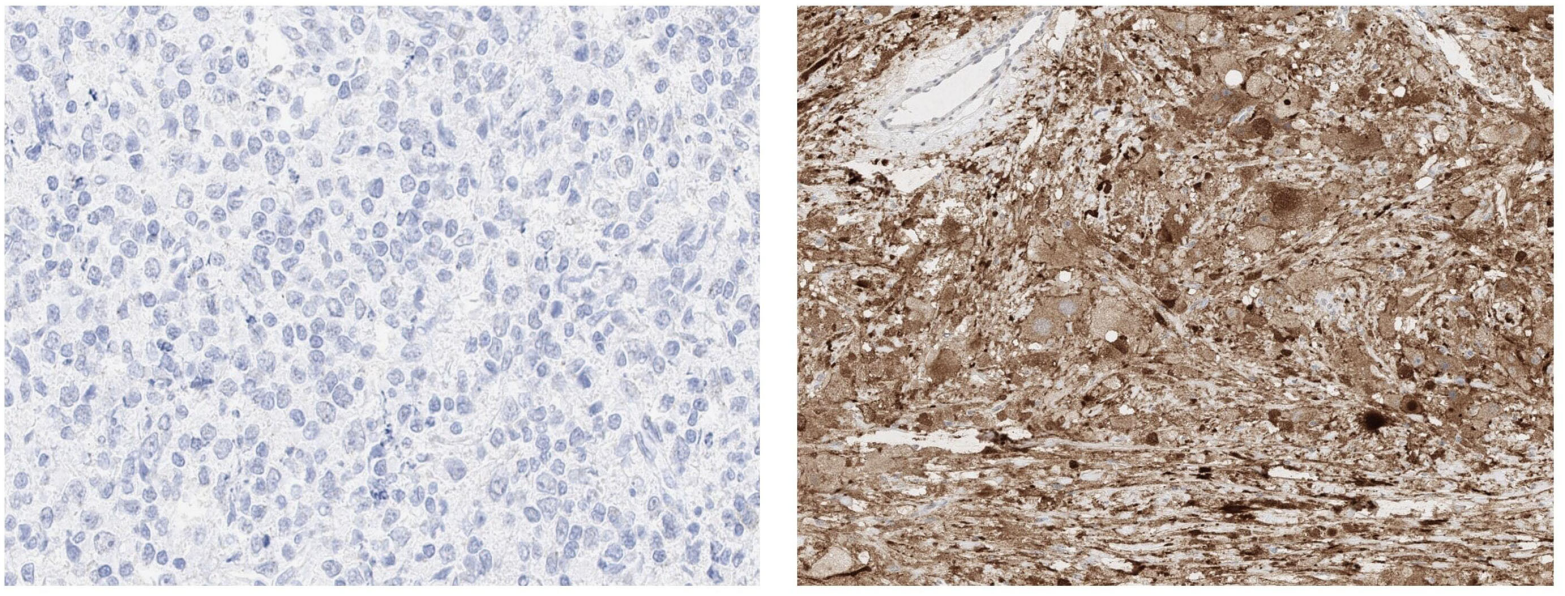
Left (400x): High magnification of spot e. No detectable positive signal. Right (100 x): Grey matter of the brain containing cell bodies with strong diffuse cytoplasmatic positivity. Of note are the negative vessels in the upper left corner.

In the TMA, 91 out of 92 (99%) DLBCL were analyzable. The one non-informative case was excluded due to unequivocal tumor tissue on the slide. Positive staining was defined as any viable staining, whether nuclear, paranuclear, cytoplasmatic, or membranous. No positive case was detected with valid positive on slide control (**Figures 2**, **3**).

### Findings by FISH

**Figure 4:**
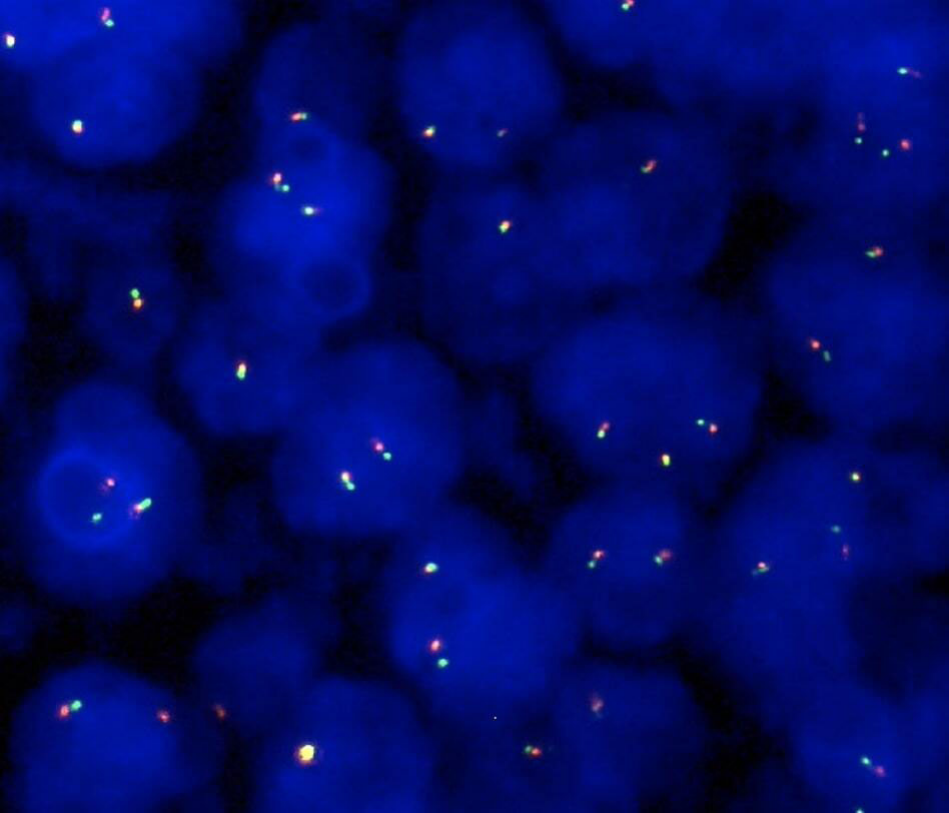
Representative example of interphase FISH finding using the NTRK2 probe (100x): Lymphoma cells lacking a translocation with two orange/green fusion signals.

For NTRK1, the hybridization quality was 2 and all spots were evaluable. For NTRK2 and NTRK3, the hybridization quality was 3 and 2, respectively, and all spots could be evaluated. All tests showed no break apart event (**Figure 4**).

## Discussion

Up to now, the role of NTRK fusion genes and protein expression in lymphoma, particularly in DLBCL, has been scarcely investigated. For this reason, we aimed to investigate whether NTRK fusion proteins are expressed in samples of DLBCL patients. By conducting a systemic immunohistochemical screening for the expression of NTRK fusion proteins and an additional FISH analysis in a large cohort of 92 consecutive DLBCL samples, an NTRK fusion protein expression was undetectable in all analyzable samples. Our results are underlined by the few data sets provided by the National Cancer Institute Genomic Data Commons data portal in which NTRK3 receptor mutations (D98N) were detectable in only one of 28 (3.6%) provided cases of mature B-cell lymphoma, whereas mutations in NTRK1 and two were not detected at all (28). One of the few analyses describing the examination of NTRK fusions in B-cell lymphoma samples is a recently published study by Witte and colleagues. The authors reported two cases of NTRK3 mutations in a subset of patients with plasmablastic lymphoma using whole-exome and RNA-sequencing (22). Similar findings were reported by Li et al. who performed a whole-exome sequencing of matched tumor tissues and blood samples from 53 patients with primary gastrointestinal diffuse large B-cell lymphoma. NTRK2 and NTRK3 were detectable in two and one sample, respectively (29). To detect NTRK fusions, several techniques are generally recommended by the ESMO Translational Research and Precision Medicine Working Group, including IHC, FISH, real-time polymerase-chain-reaction, and both RNA-based and DNA-based next-generation sequencing (13). Even though RNA-based sequencing is considered the gold standard for screening, it requires high-quality RNA for reliable sequencing and reducing the risk of false negative results (30). Moreover, next-generation sequencing remains an expansive technique that is not always widely available (13). However, IHC and FISH have been proven to have high sensitivity and specificity for detecting NTRK fusions or break aparts, respectively. False positive results are unlikely based on the limited expression of TRKA, TRKB, and TRKC in other tissues (13, 31–34). Both methods combine the advantages of rapidness, saving tumor material, and costs. Therefore a stepwise approach using IHC and/or FISH as a screening tool followed by RNA-based sequencing in cases of NTRK staining is recommended as one possible approach for the detection of NTRK fusions even in routine diagnostics (13). However, there remains a residual risk of false negative IHC results as recently described (35).

Even though no NTRK fusion protein expression and no NTRK break apart was detectable in our sample cohort, performing systemic screenings for NTRK fusions is necessary to further define the role of NTRK fusions not only in DLBCL but also in a multitude of lymphoma entities as long as the lack of reliable data exists. For this reason, additional analyses of NTRK fusions in cohorts of DLBCL by samples of similar size or larger and different detection technics are necessary to discuss our results.

## Data availability statement

The raw data supporting the conclusions of this article will be made available by the authors, without undue reservation.

## Author contributions

Conceptualization, SG, and SW. Methodology, SG, CB, and SW. Formal analysis, SG, and SW. Investigation, SG and SW. Resources, SW and CB. Data curation, SG and SW. Writing—original draft preparation, SG and SW. Writing—review and editing, JD, and CB. Visualization, SW. Supervision, JD and CB. Project administration, SG and SW. All authors contributed to the article and approved the submitted version.
